# A Review on the Role of Lactic Acid Bacteria in the Formation and Reduction of Volatile Nitrosamines in Fermented Sausages

**DOI:** 10.3390/foods12040702

**Published:** 2023-02-06

**Authors:** Selen Sallan, Zeynep Feyza Yılmaz Oral, Mükerrem Kaya

**Affiliations:** 1 Department of Food Processing, Bandırma Vocational School, Bandırma Onyedi Eylul University, 10200 Balıkesir, Türkiye; 2 Department of Food Technology, Erzurum Vocational School, Atatürk University, 25240 Erzurum, Türkiye; 3 Department of Food Engineering, Faculty of Agriculture, Atatürk University, 25240 Erzurum, Türkiye

**Keywords:** volatile nitrosamines, lactic acid bacteria, fermented sausages, biogenic amine, pH, nitrite depletion, nitrosamine reduction

## Abstract

Nitrosamines are *N*-nitroso compounds with carcinogenic, mutagenic and teratogenic properties. These compounds could be found at certain levels in fermented sausages. Fermented sausages are considered to be a suitable environment for nitrosamine formation due to acid formation and reactions such as proteolysis and lipolysis during ripening. However, lactic acid bacteria (spontaneous or starter culture), which constitute the dominant microbiota, contribute significantly to nitrosamine reduction by reducing the amount of residual nitrite through nitrite degradation, and pH decrease has an important effect on the residual nitrite amount as well. These bacteria also play an indirect role in nitrosamine reduction by suppressing the growth of bacteria that form precursors such as biogenic amines. In recent years, research interest has focused on the degradation or metabolization of nitrosamines by lactic acid bacteria. The mechanism by which these effects are seen has not been fully understood yet. In this study, the roles of lactic acid bacteria on nitrosamine formation and their indirect or direct effects on reduction of volatile nitrosamines are discussed.

## 1. Introduction

Meat plays an important role in human nutrition and is an important source of high value biological protein, minerals such as iron, zinc, selenium, and B group vitamins such as B12 [1,2]. However, due to its water activity and nutritional value, meat is an easily perishable food item. Processing techniques such as cooling, freezing, drying, curing, salting, smoking, pasteurization and sterilization are used to preserve and process meat. In addition, novel food processing techniques such as the use of ionizing radiation, ultrasound, high hydrostatic pressure, ultraviolet light, and pulsed electric fields are applied in the meat industry. A single method can be used for the preservation or processing of meat, as well as a combination of various methods [3,4,5].

Fermentation is one of the oldest and most widely used methods in food preservation, and it has an important place in the processing and preservation of meat. Fermented meat products are divided into two main groups: fermented sausages and meat products made from whole pieces. In fermented sausages, the expression “sausage ripening” is used for the changes that occur during the period from stuffing to the final product, and consists of two stages; fermentation (mainly lactic acid formation) and ageing (drying, aroma formation) [6]. The latter fermented meat products are processed from whole meat pieces, such as pastırma, dry-cured “lacón” and ham. In general, the characteristic organoleptic features of these products are established by salt, curing agents and endogenous proteolytic enzymes [6,7,8].

Fermented sausages are meat products obtained by mixing minced meat and fat in a meat grinder or cutter with various spices, sugar and other additives, filling the mix in natural or artificial intestines and ripening at a certain temperature and relative humidity [9]. The characteristic features of fermented sausages are influenced by several factors such as species and genus of animal used for lean meat and meat fat, the method used for meat chopping (cutter or meat grinder) and its degree, additives such as spices, salt, sugar, nitrite/nitrate, starter culture used, type and diameter of casing, applications such as smoking during or after fermentation, ripening conditions, slicing and packaging method [10]. Fermented sausages are classified according to various criteria considering moisture content, moisture:protein ratio (M:P), weight loss, water activity (a_w_), degree of chopping of meat and fat, and geographical region [11]. For instance, while moisture:protein ratio is considered a classification criterion in the USA, weight loss is considered in Austria, and collagen-free muscle protein (BEFFE) in Germany. However, in many countries, there is no general, clear and uniform distinction between dry and semi-dry fermented sausages, especially in traditional products. Many products with the same name could be produced as “dry” or “semi-dry” fermented sausages. In addition, unfermented-hot smoked products are also available on the market as fermented sausages [10]. The water activity in dry fermented sausages is below 0.90 and heat treatment is usually not applied in their production, while the water activity in semi-dry fermented sausages varies between 0.90 and 0.95) and they are subjected to a heat treatment process usually between 60–68 °C [12].

Nitrate (NO_3_) and/or nitrite (NO_2_) are added to fermented sausages as a curing agent. Nitrite is preferred for rapidly ripened fermented, nitrate and/or nitrite is preferred for slow ripened fermented sausages. Although nitrate is an important source of nitrite in such products ripened for a long time period, it must be reduced to nitrite in order for the expected effects from this curing agent to occur [9,13]. In addition to a_w_ and pH, nitrite is considered an important hurdle effect in fermented meat products. Nitrite plays an important role in the formation of the characteristic color, development of flavor and delay of oxidation. Nitrite is also effective in inhibiting foodborne pathogens such as *Listeria monocytogenes* and *Salmonella* in fermented sausages [14,15]. However, nitrite also plays a role in the formation of carcinogenic, mutagenic and teratogenic nitrosamines [16].

*N*-nitrosamines are recognized as important chemical hazards in fermented products [17]. Concerns about the presence of *N*-nitrosamines in meat products were raised in the early 1970s when high levels of *N*-nitrosodimethylamine (NDMA) and *N*-nitrozopyrrolidine (NPYR) were found in fried bacon. Most studies on nitrosamines were conducted in the 1970s and early 1980s, and such preventive measures as limiting the level of nitrites added and using antioxidants such as ascorbate in the production of meat products were highlighted to control this contamination [18]. pH and secondary amines are the most important factors besides nitrite and residual nitrite that are involved in the formation of nitrosamines in fermented sausages. Another important factor is the cooking method and cooking time [19,20,21]. Fermented sausages are safe, ready-to-eat products [22]. On the other hand, some fermented sausage types are cooked before consumption due to consumption habits [21,23,24,25]. Since acidification during fermentation, and proteolysis and lipolysis reactions during drying/ripening turns fermented sausages into a suitable environment for nitrosamine formation, cooking prior to consumption significantly increases the risk of nitrosamines [24,25]. On the other hand, nitrosamines may as well be detected in fermented sausages that were not subjected to any heat treatment, and the nitrosamine content may vary greatly. Total nitrosamine level is reported to be under 5.5 μg/kg, as well as at lower levels in meat products [17]. *N*-nitrosodimethylamine (NDMA), *N*-nitrozopyrrolidine (NPYR), and *N*-nitrozopiperidine (NPIP) are widely identified in fermented sausages [26]. NDMA is defined as a probable carcinogenic compound in the 2A group, whereas NPIP and NPYR are possible carcinogenic compounds (2B) [27]. Besides that, *N*-nitrosomorpholine (NMOR), *N*-Nitrosodiethylamine (NDEA), *N*-Nitrosodibutylamine (NDBA), *N*-nitrosomethylethylamine (NMEA) are some other nitrosamines that can be seen in fermented sausages [18,21].

Various strategies have been developed to reduce the formation of nitrosamines. Many studies have been conducted on the possibilities of using compounds such as ascorbic acid and polyphenols as inhibitors in the formation of nitrosamines [24,25,28,29,30]. In addition, studies were conducted to examine the effect of nitrite levels lower than the legal limit of 150 mg/kg on nitrosamine formation [28,31]. On the other hand, to eliminate the risk of nitrosamine formation in sausages, the use of nitrate-rich vegetables as sources of nitrite has come to the fore. It was reported that celery juice and celery powder are more likely to be used as a natural source of nitrates and/or nitrites, since they do not negatively affect the flavor of processed meat products [32]. Other alternatives are bacteriocins, bioprotective cultures, high hydrostatic pressure, organic acids such as lactate, and acetate. However, it is not possible to provide all the functions of nitrite with these applications alone [32,33,34,35]. Therefore, it is recommended to use hurdle technologies in the curing process. [32]. On the other hand, studies on the effects of indigenous lactic acid bacteria strains on nitrosamine formation, directly or indirectly, have been conducted in vitro and in vivo [36,37,38,39]. In the present study, studies on the formation of nitrosamines in fermented sausages and the role of lactic acid bacteria in this formation, the presence of nitrosamines in these products, and the reduction of nitrosamines by lactic acid bacteria were critically reviewed and recommendations were made.

## 2. Lactic Acid Bacteria and Nitrosamine Formation in Fermented Sausages

Traditional dry fermented sausages rely on spontaneous fermentation by indigenous microbiota that can originate from the raw material or from the environment. Such microbiota is called “house flora” and is composed of technologically important microorganisms during ripening, as well as of spoilage microorganisms that can negatively affect the properties of the final product, and may also include some pathogenic microorganisms [40,41]. Excellent fermented sausages are reported to be manufactured without addition of starter cultures or re-inoculation (back slopping) with finished sausages [6].

Lactic acid bacteria and Gram (+) catalase positive cocci are two groups of microorganisms that are technologically important during the fermentation and ripening of fermented sausages. Among the identified lactic acid bacteria, *Latilactobacillus sakei*, *L. Latilactobacillus curvatus*, *Lactiplantibacillus plantarum* are the most commonly identified species [11,42,43,44,45,46]. *Pediococcus acidilactici*, P. *pentosaceus*, *Lactiplantibacillus pentosus* and *L. plantarum*, which are used as starter cultures in fermented meat products, are isolated less commonly when compared to *L. sakei* or *L. curvatus*, due to the fact that their competitiveness is low in the majority of traditionally-produced fermented sausages. These species cause considerable acidification in the first days of the fermentation, but they may not be able to prevent the development of non-starter lactic acid bacteria that may demonstrate undesirable effects in the final product. On the other hand, *L. plantarum* may also cause high acidity in the final product [47]. *Pediococcus* is preferred to lactobacilli in those products which are rapidly ripened at high temperatures. Moreover, as the ripening period proceeds, the number of *Pediococcus* decreases [48].

*Lactobacillus* and *Pediococcus* species are used as lactic starter cultures in fermented meat products. *L. sakei* and *L. curvatus* species show good growth at fermentation temperatures of 20–24 °C and maintain their vitality significantly during ripening. *L. plantarum* and *Pediococcus* spp dominate the environment at higher fermentation temperatures [13]. The most important role of lactic acid bacteria in fermented meat products is to ensure product safety by forming acid. In addition, these bacteria are also effective in aroma and texture formation, and color development by means of acid production. As a result of lactic acid accumulation during fermentation, pH approaches the isoelectric point of myofibrillar proteins and facilitates the drying of the product, making a significant contribution to texture development [13]. The pH decrease caused by lactic acid bacteria also positively affects product preservation [6,13]. The fermentation is the most critical stage in fermented meat products, the pH drop with acid formation is important for the prevention and control of the growth of foodborne pathogens such as *Listeria monocytogenes*, *Staphylococcus aureus*, and *Salmonella* [49,50,51].

The pH drop caused by lactic acid bacteria is also an important factor in the conversion of nitrite to nitric oxide. As the pH of the meat decreases, the concentration of nitrous acid increases, and therefore the nitrite concentration changes as a function of pH level [52]. Nitrite can transform into various compounds including nitrous acid, nitric oxide, and nitrate [53]. While 1–15% of nitrite is bound to lipids, 1–5% of nitrite is lost as nitric oxide gas. This curing agent is transformed to nitrate with 1–10% and 5–20% of nitrite remains free. In addition, 1–15% of this compound reacts with sulfhydryl compounds, mainly with peptides and free amino acids, and 5–15% with myoglobin. Also, 20–30% of nitrite is bound to other proteins [54]. Nitrite, which is a multifunctional additive, can also participate in nitrosation reactions [53]. *N*-nitrosamines, which are formed by the electrophilic substitution reaction between the nitrozonium cation (NO^+^) provided by the nitrosation agent (Y-NO) and the amine nitrogen, are compounds associated with carcinogenic, mutagenic and teratogenic properties [55,56]. The presence of amines and acidic pH conditions are prerequisites for nitrosamine formation. The pH drop caused by lactic acid bacteria is an important factor for nitrosamine formation [57,58]. The pH must be low enough for the formation of NO^+^, which is necessary for the nitrosation reaction [57]. In general, meat products have a pH between 5.5 and 6.2, while dry fermented sausages have a pH in the range of 4.5–5.5 [59]. It was reported that *N*-nitrosamine formation may occur more easily in dry fermented sausages as the pH of the product approaches the optimum pH (pH 3.5) of the nitrosation reaction [59,60]. Indeed, Sallan et al. (2020) reported the combination of starter culture (low pH) and high nitrite was effective in the formation of NPYR [25].

Lipolysis, lipid oxidation and proteolysis occurring during ripening in fermented sausages are important phenomena for the flavor and aroma [61,62]. Protein and lipid degradation products generated during ripening (fermentation/drying) can be significant sources of nitrosamine formation [16]. Lactic acid bacteria demonstrate weak proteolytic and lipolytic activity under fermented sausage conditions [6] and make a partial contribution to flavor development [63]. Coagulase-negative staphylococci (CNS), an important part of the microbiota of fermented sausage, are effective in the proteolysis and lipolysis that occur during fermentation and drying [9]. Catalase-positive cocci (mainly CNS), used as starter cultures in fermented sausages and found spontaneously, are more effective at proteolysis than lactic acid bacteria. Likewise, these microorganisms are much more active in lipolysis than lactic acid bacteria. In other words, lactic acid bacteria have weak lipolytic activity [6,9]. It was also reported by Demeyer et al. (2000), that bacterial lipases showed low lipolytic activity under conditions of fermented sausage production [64]. Muscle lipases and phospholipases are essentially responsible for lipolysis in meat products [65]. However, this is a strain-dependent activity and should be controlled accurately to ensure the quality of the end product [42].

Lactic acid bacteria show low proteolytic activity, but the pH decrease caused by these bacteria contributes to proteolysis [66,67]. On the other hand, it was reported that some strains of *L. sakei,* *L. curvatus* and *L. plantarum* have several peptidase activities such as leucine and aminopeptidase [43,67,68,69,70,71]. However, free amino acids formed by proteolysis can indirectly play a role as precursors in the formation of nitrosamines. For example, free proline can take part in the reaction as a precursor in the formation of NPYR. However, it is reported that biogenic amines formed from free amino acids by the decarboxylase activity of microorganisms can be nitrosatable amine precursors in meat products [16]. The most frequently detected biogenic amines in fermented sausages are tyramine, putrescine and cadaverine, which are each composed of tyrosine, ornithine and lysine [59]. Putrescine, spermidine and spermine can be effective in the formation of NPYR [72]. Lysine and cadaverine, formed as a result of the bacterial decarboxylase activity of this amino acid, can also be converted to NPIP in a variety of ways. Cadaverine is converted to piperidine by deamination and cyclization reactions prior to nitrosation. Piperidine, on the other hand, can react with the nitrosating agent to form NPIP [73,74,75]. On the other hand, it is emphasized that there may not be a direct link between nitrosamine contamination and biogenic amine contents of dry fermented sausages [18,59]. The availability of biogenic amine and protein degradation products are thought to be an important source of amine precursors. Secondary amines such as dimethylamine, which are protein degradation products, can be directly involved in the nitrosation reaction [76]. However, biogenic amines such as spermidine, spermine, cadaverine and putrescine containing primary amine groups must be converted to nitrosable secondary amines by deamination and cyclization reactions [73,77].

## 3. Occurrence of Volatile Nitrosamines in Fermented Sausages

Nitrosamines are divided into two groups as volatile and non-volatile. Volatile nitrosamines are relatively nonpolar and low molecular weight compounds [78]. Volatile nitrosamines include *N*-nitrosodiethylamine (NDEA), *N*-nitrosodimethylamine (NDMA), *N*-nitrosopiperidine (NPIP), *N*-nitrozopyrrolidine (NPYR), *N*-nitrosodibutylamine (NDBA), *N*-nitrosomorpholine (NMOR), *N*-nitrozodipropylamine (NDPA), and *N*-nitrosodibenzylamine (NDBzA) [79].

Non-volatile nitrosamines are *N*-nitrosodiphenylamine (NDPhA), *N*-nitrosomethylaniline (NMA), *N*-nitroso-2-methyl-thiazolidine-4-carboxylic acid (NMTCA), *N*-nitrosoproline, *N*-nitrosohydroxyproline, *N*-nitrososarcosine, *N*-nitrosothiazolidine, *N*-nitrosothiazolidine-4-carboxylic acid, *N*-nitrozooxazolidine-4-carboxylic acid, *N*-nitroso-5-methyloxazoldine-4-carboxylic acid, and *N*-nitroso-2-(hydroxymethyl) thiazolidine-4-carboxylic acid [79].

In cured meat products, the nitrosamine level can vary significantly depending on the product and type of nitrosamine. While values lower than 5 μg/kg are usually detected in these products, nitrosamine level may be as high as 20 μg/kg. Nitrosamine content in fermented sausages, which are cured meat products, varies significantly [17,54]. Factors such as raw material, spice, ingoing and residual nitrite, presence of catalyst and inhibitors, fermentation and drying (ripening) conditions, pH and a_w_ value of the final product are effective in this variation [16]. NDEA, which was evaluated in the 2A group by IARC, was detected at a lower rate in fermented products than NPIP, NDMA and NPYR (Table 1). However, the amount of NDEA in Mettwurst (spreadable fermented sausage) and salami was found to be 7.5 μg/kg and 12.0 μg/kg, respectively [80]. In later studies, the NDEA levels for salami and sausage were found to be below 1 μg/kg [19,31,81]. NDEA is stated to consist of the amino acid alanine. It is also frequently suggested that this nitrosamine is involved in the migration of amine precursors from packaging material [16]. Another nitrosamine that has been linked to the migration of packaging-derived amine precursors is NDBA [16,82,83,84]. NDBA is a rare nitrosamine in meat products, like NDEA and it could be detected at very low levels in meat products [19,28,81,85]. However, Gavinelli et al. (1988) detected 50.12 μg/kg of NDBA in salami [86]. Another nitrosamine contaminated from packaging material is NMOR. The frequency and occurrence level of NMOR is very low (Table 1).

The level of NDMA, which is one of the commonly determined nitrosamines in fermented sausages, varies quite significantly. The precursor of NDMA is dimethylamine, a secondary amine. Another precursor of NDMA is putrescine. Fermented sausages are protein-rich foods, and as a result of proteolysis during ripening, amines are formed by endogenous or microbial enzymes, which may cause an increase in NDMA. In addition, protein oxidation can increase the amine content in these products, which are complex food systems [87]. Furthermore, the pH and a_w_ values of fermented sausages indirectly affect the formation of NDMA through affecting the growth of some microorganisms with aminoacid decarboxylase activity [88]. While the pH drop during fermentation in fermented sausages increases decarboxylase activity, it can inhibit the growth of some microorganisms (especially *Enterobactericeaea* and *micrococci*/*staphylococci*) with this activity [89]. One of the most effective factors in the formation of NDMA is the ingoing nitrite level. NDMA level increases in parallel with increasing nitrite level in fermented sausages [25,38,72]. Herrmann et al. (2015a) suggest that there is no significant correlation between nitrite level and nitrosamine content in both dried sausages and dried-fried sausages [28]. NDMA level was generally found to be below 1 μg/kg in fermented sausages [19,80,81,90,91]. In some studies, however, the NDMA level was determined to be below 4 μg/kg [80,91,92,93]. Gavinelli et al. (1988) found the NDMA content in the salami sample to be 7.76 μg/kg [86]. On the other hand, Herrmann et al. (2015b) determined the mean NDMA value as 1.6 μg/kg in samples from the Danish market and 2.6 μg/kg in Belgian samples [31] (Table 1). The same researchers detected NDMA at the level of 4.0 μg/kg in one sample (from Belgium) and 7.2 µg/kg in another sample. From these results, it can be said that NDMA is generally below 5.0 μg/kg in fermented sausages. One of the most effective factors in the formation of NDMA in meat and meat products is heat treatment [18]. Although fermented sausages are reliable ready-to-eat products, in some countries these products are consumed after cooking [21,23,24,25].

Spice is an important ingredient in fermented sausages, and some products are even characterized by spice. Black pepper, which is included in the formulation of many fermented sausages, plays a role in the formation of nitrozopiperidine (NPIP) since it contains piperine and piperidine [18]. NPIP was detected to be below 1 μg/kg in many products [19,80,86] (Table 1). It has been reported that the NPIP content varies between 0.32–0.95 μg/kg in sucuk containing up to 1% black pepper in the formulation [91]. However, in previous studies, maximum values such as 2.71 and 3.07 μg/kg were determined for NPIP [81,92]. On the other hand, higher NPIP values were reported in heat-treated sausage containing a significant amount of black pepper in the formulation [93] (Table 1). Herrmann et al. (2015b) stated that heat treatment has a positive effect on the NPIP level [31]. Similarly, Drabik-Markiewicz et al. (2011) reported that intense heat treatment and high nitrite concentration have a significant effect on the increase in NPIP, and the effect of heat treatment is relatively greater [72]. Fermented sausages are good environments for biogenic amine formation. During the ripening and storage of fermented sausages, biogenic amines such as tyramine, putrescine and cadaverine are formed through microbial decarboxylation of tyrosine, ornithine and lysine amino acids, respectively [55]. Cadaverine turns into piperidine and plays a role in the formation of NPIP with the effect of heat treatment [94].

NPYR is one of the nitrosamines frequently detected in fermented sausages as NDMA. The major source of this nitrosamine is pyrrolidine. Many spices are also good sources of this compound. In addition, putrescine is also effective in the formation of NPYR [16]. While maximum values ranging from 2.0 to 4.0 μg/kg were detected in fermented sausages containing NPYR [31,90,92], values below 1.0 μg/kg were observed in some other studies [19,81]. However, in another study, 1.65–7.29 μg/kg values were found for NPYR in heat-treated sucuk [93] (Table 1).

**Table 1:** Occurrence of volatile nitrosamines in fermented sausages.

Fermented Sausage	Number of Samples Analyzed	Number of Samples with Nitrosamine	Detected NAs	NAs Level (μg/kg)		References
				Range	Mean	
Raw ripened sausages (Salami, German sausages etc.)	15	8	NDMA	0.1–0.6	-	[90]
	15	3	NPIP	1.0–5.0	-	
	15	1	NPYR	3.0	3.0	
German sausage (Mettwurst), pate (non-smoked)	10	10	NDMA	nd–0.84	0.26	[80]
	10	8	NDEA	0.02–7.5	1.1	
	10	8	NDBA	nd–1.3	0.26	
	10	6	NPIP	nd–0.41	0.06	
	10	4	NMOR	nd–1.7	0.24	
Salami	4	4	NDMA	0.59–7.76	5.35	[86]
	4	4	NDEA	nd–4.04	0.35	
	4	4	NDBA	0.73–50.12	19.71	
	4	4	NPIP	nd–0.38	nd	
Salami	4	4	NDMA	nd–2.1	0.45	[80]
	4	4	NDEA	1.5–12	4.6	
	4	4	NDBA	0.29–2.0	0.56	
	4	3	NPIP	nd–0.58	0.17	
Salami	10	10	NDMA	-	0.84	[19]
	10	10	NDEA	-	0.67	
	10	10	NPYR	-	0.93	
	10	10	NPIP	-	0.64	
	10	10	NDBA	-	0.84	
Salami (Danish samples)	24	15	NDMA	-	1.6	[31]
	24	1	NMOR	-	0.5	
	24	10	NPYR	-	2.1	
	24	6	NDEA	-	0.3	
	24	5	NPIP	-	0.1	
Salami (Belgian samples)	9	5	NDMA	-	2.6	[31]
	9	1	NMOR	-	0.5	
	9	7	NPYR	-	2.7	
	9	7	NPIP	-	0.3	
Sucuk	10	10	NDMA	2.21 ± 0.82	-	[92]
	10	10	NMEA	1.16 ± 0.39	-	
	10	10	NDEA	2.25 ± 0.70	-	
	10	10	NPYR	3.84 ± 0.88	-	
	10	10	NMOR	0.21 ± 0.07	-	
	10	10	NPIP	3.07 ± 0.89	-	
	10	10	NDBA	1.25 ± 0.40	-	
	10	10	NEBA	0.53 ± 0.41	-	
Sucuk	6	5	NDMA	0.11–0.78	-	[81]
	6	4	NDEA	0.10–0.95	-	
	6	5	NDPA	0.27–1.35	-	
	6	6	NPYR	0.11–1.36	-	
	6	6	NPIP	0.16–2.71	-	
	6	4	NDBA	0.15–1.68	-	
Sucuk	30	7	NDMA	0.40–0.81	0.52	[91]
	30	3	NMEA	0.43–0.48	0.45	
	30	15	NPIP	0.32–0.95	0.61	
Heat-treated sucuk	30	30	NDMA	1.71–3.57	-	[93]
	30	30	NPYR	1.65–7.29	-	
	30	30	NPIP	5.19–16.40	-	

Dash (-) indicates that values are not reported or not detected; nd: not detected.

## 4. Possibilities for Nitrosamine Reduction by Lactic Acid Bacteria in Fermented Sausages

### 4.1. Inhibition of Biogenic Amine Positive Microorganisms

Biogenic amines, which are formed by bacterial decarboxylation of free amino acids in fermented sausages, can be indirectly involved in the formation of nitrosamines. Studies have shown that some biogenic amines can play a precursor role [72,77,94,95,96]. These amines could be a good source of secondary amines that react with nitrite [97]. Therefore, it is important to prevent contamination with microorganisms having decarboxylase activity, namely biogenic amine-positive microorganisms, and also to prevent the growth of these microorganisms during fermentation for nitrosamine reduction.

Both Gram (−) and Gram (+) bacteria that are spontaneously present in fermented sausages can form biogenic amines. Members of the *Enterobacteriaceae* family and *Pseudomonas* spp are known as major producers of cadaverine, histamine and putrescine [98]. In fermented sausages, the early stage of ripening, the fermentation stage, is important for many reactions. Depending on the rate and degree of acidification in sausage fermentation, undesirable microbiota are also suppressed. The growth of *Enterobacteriaceae* is inhibited and thus the formation of biogenic amines is limited [9]. Very high levels of biogenic amines in fermented sausages are reported to indicate high levels of undesirable microbiota such as *Enterobacteriaceae*. This situation may also be evaluated as an indicator of low raw material quality and/or hygienic and technological rules not being followed during production [99]. Histidine, lysine, ornithine and tyrosine decarboxylase activities were detected in many *Enterobacteriaceae* family members isolated from fermented sausages [100]. In fermented sausages, especially in the presence of lactic starter cultures, the number of *Enterobacteriaceae* decreases as the ripening period progresses [9,43,75]. Similarly, the growth of *Micrococci* and *Staphylococci* with decarboxylase activity is prevented by acidification during fermentation. These microorganisms are technologically important due to aroma formation, nitrate reductase activity and catalase activities in fermented sausages. Therefore, *Staphylococcus xylosus,* S. *carnosus, Kocuria varians* are added as a starter culture together with lactic acid bacteria in fermented sausages (6). However, micrococci and CNS to be used as starter cultures should also be tested for decarboxylase activity.

Biogenic amine levels in fermented products vary greatly depending on the raw material, fermentation conditions and microbiota. The literature is ripe with studies on the occurrence of biogenic amines in fermented meat products. Tyramine, histamine, phenylethylamine, tryptamine, putrescine, cadaverine, spermine and spermidine have been detected in a large number of analyzed products [42,100,101,102,103,104,105,106,107,108,109,110]. That being said, tyramine, putrescine and cadaverine are the main determined biogenic amines in fermented sausages. While the presence of putrescine and tyramine in fermented sausages is associated with the activity of lactic acid bacteria, it is stated that cadaverine is caused by insufficient hygienic measures and high microbial load of the raw material used in production [99]. Although the aminogenic potential of lactic acid bacteria is low, it can contribute to biogenic amine accumulation. Some lactic acid bacteria have amino acid decarboxylase activity and can form histamine, cadaverine and putrescine, while they are particularly effective in terms of tyramine formation. Therefore, the selection of strains used as starter cultures in fermented sausages is very important [98]. On the other hand, lactic acid bacteria are considered microorganisms that are responsible for the formation of biogenic amines in fermented meat products. A high *Lactobacillus* number is reported to have a significant impact on histamine formation [98,100,111]. While tyramine-producing strains have been attributed to *Carnobacterium*, *Latilactobacillus curvatus*, and *L. plantarum, Micrococcaceae* and *Latilactobacillus sake* have been reported to play no role in tyramine formation [112]. It is stated that *Enterococcus faecalis* and E. *faecium* species, which belong to the lactic acid bacteria group, also produce high levels of tyramine. *L. curvatus* strains are reported to play a role in the formation of phenylethylamine, tryptamine, putrescine and cadaverine as well as tyramine [99]. Yet, some studies have reported that amine-negative *L. sakei* and *Pediococus pentosaceus* inhibit the formation of biogenic amines [100]. The most important factor is low pH in terms of the inhibition of the growth of amine decarboxylase-positive microorganisms. As a matter of fact, it has been demonstrated in many studies on fermented sausages in the literature, that the use of starter culture is important in terms of biogenic amine control, as well as hygienic raw materials [110,113,114,115]. Moreover, Xiao et al. (2018) reported that *Lactiplantibacillus pentosus* increases the competitiveness of the desired microbiota in dry fermented sausage production and also contributes to nitrosamine reduction by inhibiting the growth of microorganisms with decarboxylase activity and nitrate reductase activity [88].

It was stated that putrescine, spermidine and spermine, which are biogenic amines determined in fermented sausages, can be effective in the formation of NPYR [72]. In another study, it has been reported that spermine and spermidine may play a role in the formation of *N*-nitrosopyrrolidine (NPYR), and cadaverine or piperidine may play a role in the formation of NPIP [116]. Cadaverine, which is formed as a result of lysine and the bacterial decarboxylase activity of this amino acid, can also be converted to NPIP in different ways. Cadaverine is converted to piperidine by deamination and cyclization reactions before the nitrosation reaction. Piperidine, on the other hand, can react with the nitrosation agent to form NPIP [73,74,75]. On the other hand, it is also emphasized that there may not be a direct link between nitrosamine contamination of biogenic amines and biogenic amine contents of dry fermented sausages [16,18].

### 4.2. Effect of Nitrite Depletion/Degradation on Nitrosamine Reduction

The nitrite level of meat products is an important factor in nitrosamine formation [117]. Therefore, the nitrite content to be used in meat products is limited to 150 mg/kg in the related legislation of the European Union due to the nitrosamine risk [118]. However, it is noted that the risk of nitrosamines in meat products still remains [16,24,25,93].

The decrease in pH during fermentation in fermented sausages accelerates the conversion of nitrite to nitric oxide and thus causes a significant decrease in the residual nitrite level. Considering the relationship between the residual nitrite level and the formation of some nitrosamines, it turns out that lactic acid bacteria exert an indirect inhibitory effect by lowering the residual nitrite level. As a matter of fact, it is stated that the amount of residual nitrite is an important factor in many nitrosamines [119]. In addition, some lactic acid bacteria have nitrite reductase and heme-independent nitrite reductase and are converted to NO, NO_2_ or N_2_O through these enzymes under anaerobic conditions and reduce the residual nitrite level [120]. A rapid pH drop during fermentation can result in faster nitrite depletion [51,120,121,122,123]. In a study on Chinese fermented sausage, it was reported that *Lactiplantibacillus plantarum*, which has nitrite and nitrate reductase activity, reduced the amount of residual nitrite [124]. Similarly, Wang et al. (2013) reported that the nitrite level decreased from 100 ppm to 9.6 ppm in fermented sausage in which *Latilactobacillus sakei* was used as starter culture [120]. The same investigators reported that this decrease was slower in spontaneous fermentation. It was also stated that *L. plantarum* CMRC6 and *L. sakei* CMRC15 strains each have very good nitrite reductase capacity in a study [122]. In this respect, since residual nitrite plays an important role in the formation of nitrosamines, nitrite reductase activity of lactic acid bacteria is thought to be effective in detecting lower levels of nitrosamines. As a matter of fact, the nitrite reductase activity of lactic acid bacteria is considered as another criterion in the selection of starter cultures [120]. Besides the effect of pH, nitrite depletion depends on such factors as raw meat type, initial nitrite levels, storage and processing conditions, and the availability of reducing agents [125]. Additionally, sodium ascorbate, used as a curing accelerator in fermented sausages, increases nitric oxide production, resulting in lower residual nitrite levels. Furthermore, ascorbate reduces Fe^+3^ in metmyoglobin to Fe^+2^ in deoxymyoglobin in cured meats (pH: 5.0–6.0) [126]. For this reason, ascorbate is widely used to reduce the nitrite level used in meat products and reduce the residual amount of nitrite.

### 4.3. Adsorption and Degradation of Nitrosamines by Lactic Acid Bacteria

In fermented sausages, lactic acid bacteria indirectly contribute to nitrosamine reduction by inhibiting decarboxylase-positive microorganisms and reducing residual nitrite levels. Approaches that use of lactic acid bacteria, which constitute the dominant microbiota in fermented sausages, can be effective in nitrosamine reduction by direct degradation and adsorption and have been frequently highlighted in recent years. One of the first studies in this regard involves a traditional fermented vegetable product called kimchi. In this study, the effects of *Leuconostoc carnosum*, *Le. mesenteroides*, *Lactiplantibacillus plantarum* and *Latilactobacillus sakei* strains on NDMA and its precursors were examined using the model system during kimchi production and digestion. In addition, the ability of strains to deplete NDMA and nitrite was investigated in the study. The researchers found that the four strains directly depleted NDMA in the broth and reduced nitrite levels, with this effect being greater in *L. plantarum* and *L. sakei*. It was also reported that there is a significant reduction in the levels of NDMA and its precursors (nitrite, dimethylamine and biogenic amine) compared to the control group in the presence of strains in kimchi production and that the reduction occurs in relation to the bacterial load. As a result, probiotic bacteria were reported to be the cause of the significant decrease in NDMA occurring in kimchi, possibly via direct degradation and inhibition of NDMA formation (37). In a study on indigenous strains (*L. plantarum* 120, *Saccharomyces cerevisiae* 2018 and *Staphylococcus xylosus* 135) isolated from Chinese traditional fermented fish products, it was reported that three strains directly degraded NDMA and *L. plantarum* 120 was observed to have the highest degradation rate [39].

Investigations on nitrosamine degradation of lactic acid bacteria strains isolated from fermented sausages carried out in fermented sausages in vitro are listed in Table 2. In a study by Nowak et al. (2014), the detoxifying effect of four probiotic strains of Lactobacillus (*Lacticaseibacillus rhamnosus* ŁOCK 0900, *L. rhamnosus* ŁOCK 0908, *Lacticaseibacillus casei* ŁOCK 0919, and *Levilactobacillus brevis* 0945) on NDMA under different culture conditions (24 h for MRS, 168 h for phosphate buffer and 168 h for modified MRS-N) were investigated and it was reported that the NDMA level decreased up to 50% depending on the pH, strain, NDMA concentration, incubation time, and culture conditions. It was also noted that the most effective strain in reducing the concentration and genotoxicity of NDMA was *L. brevis* 0945, and that these results could be related to adsorption or metabolism [127].

In a study examining the effects of *Lactiplantibacillus pentosus* R1, *Latilactobacillus curvatus* R5 and *L. sake* L6 strains on N-nitrosamine formation and quality characteristics in Harbin dry sausage, *L. curvatus* showed a highly inhibitory effect on the four identified nitrosamines (*N*-nitrosodiethylamine (NDEA), *N*-nitrosodipropylamine (NDPA), *N*-nitrosodiphenylamine (NDPhA), and *N*-nitrosopiperidine (NPIP)), whereas *L. pentosus* R1 was only effective on NDPA and NDphA. These strains have also been reported to significantly reduce residual nitrite levels compared to the control. As a result, it was emphasized that the strains examined could be used as starter cultures to improve quality traits and reduce nitrosamine accumulation in Harbin dry fermented sausage, and among the strains examined, *L. curvatus* R5 gave the best performance in preventing nitrosamine formation [38].

In another study conducted on a Chinese dry fermented sausage type, it was reported that the nitrosamine content, residual nitrite, carbonyl content, total volatile basic nitrogen, pH and water activity were lower compared to the control at the end of ripening of sausages produced with *L. pentosus.* It was determined that *L. pentosus* reduced NDMA, NDEA, NPYR and total nitrosamine contents in fermented sausages by 13.8%, 1.89%, 15.55% and 11.72%, respectively, at the end of ripening. In addition, it was reported that the nitrosamine degradation rate of this strain in peptone water medium was 4.60%, 4.87% and 16.35% on days 0, 10 and 20, respectively, and the strain showed nitrite depletion ability in MRS broth. As a result, it was stated that *L. pentosus* can directly degrade nitrosamines in dry fermented sausages, indirectly reduce their precursors or inhibit nitrosamine formation under low pH and water activity conditions, which is associated with higher microbiological quality [88].

A model system was prepared using NDMA, its precursors, and ferrous sulfate medium in a study conducted to clarify the NDMA reduction mechanism of *L. pentosus* R3 sourced from Chinese dried sausage. It was reported in the study that *L. pentosus* R3 significantly reduced the NDMA content and the levels of dimethylamine and putrescine. In the same study, it was also reported that nitrite was degraded to nitrosonium cation by acidolysis, which may promote the NDMA formation, and *L. pentosus* R3 directly degraded NDMA and showed antioxidant activity. Consequently, three ways were suggested for the strain to reduce NDMA; inhibition of the formation reaction, a decrease in precursors (DMA and putrescine) and direct degradation [128].

Fermented sausages are generally divided into two groups, dry and semi-dry. The water activity in dry fermented sausages is below 0.90 and heat treatment is generally not applied in their production. The water activity in semi-dry fermented sausages varies between 0.90 and 0.95, and a heat treatment is usually between 60–68°C [129]. However, semi-dry fermented sausages that are subjected to more intense heat treatment can also be produced. In this type of product, fermentation, drying and cooking are the main processes and these products are called fermented cooked sausages [130]. The effect of *L. pentosus* R3 on NDMA during production was investigated in a study on a fermented cooked sausage produced by applying fermentation (85% RH at 25°C), drying (25°C, 65% RH) and cooking (at 100 °C for 15 min). Sausages produced using this strain were reported to yield lower dimethylamine and carbonyl content, thiobarbituric acid reactive substances value, and content of non-haem iron values than the control group. Researchers have reported that *L. pentosus* R3 can directly degrade NDMA, reduce its precursors, reduce oxidation, and indirectly affect NDMA by causing a reduction in non-heme iron levels. Furthermore, they stated that low pH and water activity levels inhibit NDMA by improving the microbial quality of the sausage [131].

**Table 2:** Effects of some autochthonous strains on nitrosamine reduction in fermented sausage and model systems.

Lactic Acid Bacteria Strains	Source of Strains	Environment	Nitrosamines	Mechanism	Reference
*Lacticaseibacillus rhamnosus* ŁOCK 0900 *L. rhamnosus* ŁOCK 0908 *Lacticaseibacillus casei* ŁOCK 0919 *Levilactobacillus brevis* 0945	Human Human Human Plant	In Vitro	NDMA	Adsorption or metabolism	[127]
*Lactiplantibacillus pentosus* R1 *Latilactobacillus curvatus* R5 *Latilactobacillus sakei* L6	Harbin dry sausage	Harbin dry sausage	NDEA, NDPA, NDPhA, NPIP (*L. curvatus* R5) NDPA, NDPhA (*L. pentosus* R1)	Inhibit nitrosamine accumulation	[38]
*Lactiplantibacillus pentosus* R3	Chinese dry sausage	Chinese dry sausage In vitro	NPYR, NDMA, NDEA, Total nitrosamines	Directly by degrading nitrosamines Indirectly by decreasing their precursors (nitrite, amines)	[88]
*L. pentosus* R3	Chinese dry sausage	In vitro	NDMA	Direct degradation Decreasing the precursors of NDMA	[128]
*L. pentosus* R3	Chinese dry sausage	Fermented cooked sausage	NDMA	Direct degradation Indirect reduction of its precursors Weakened oxidation Decrease in the non-haem iron content	[131]

## 5. Conclusions

Fermented sausages are cured meat products that attract attention with their typical characteristic aroma. In almost all regions there are many types of products made by both traditional methods and industrial production. Fermentation, the most critical phase of these products, takes place by spontaneous lactic acid bacteria or lactic acid starter cultures. In addition, nitrate and/or nitrite is used as a curing agent in these products for product safety, characteristic cured flavor and cured color. Because of this curing agent, these products have long been in the spotlight due to the risk of nitrosamines. Various strategies have been developed to reduce nitrosamine levels due to their carcinogenic, teratogenic and mutagenic properties. In this regard, the nitrite content used was limited firstly, meanwhile, the use of reducing compounds such as ascorbic acid and sodium ascorbate have also became widespread in fermented sausages. In addition, the role of lactic acid bacteria in reducing the formation and level of nitrosamines is also being investigated. Lactic acid bacteria reduce the residual nitrite level by forming acid and thus contribute to nitrosamine reduction. Furthermore, these microorganisms inhibit the growth of biogenic amine-forming microorganisms by dominating the environment during fermentation since biogenic amines could be the precursors of nitrosamines. The nitrite depletion ability of lactic acid bacteria can also be significantly effective in lowering nitrosamine levels. In addition to all these, it is stated that lactic acid bacteria can prevent nitrosamine accumulation through direct adsorption or metabolism. However, new research is still needed to fully elucidate its mechanism.

## Data Availability

No new data were created or analyzed in this study. Data sharing is not applicable to this article.
